# Hepatocellular Carcinoma Segmentation on MRI: Five-Fold ATLAS Benchmarking, Phase-Wise External Stress Testing, and Multiphasic OpenSwissHCC Ablation

**DOI:** 10.3390/tomography12090133

**Published:** 2026-09-15

**Authors:** George Sgourakis

**Affiliations:** 1 East Lancashire Hospitals NHS Trust, Royal Blackburn Teaching Hospital, Haslingden Road, Blackburn BB2 3HH, UK; georgios.sgourakis@elht.nhs.uk; 2 School of Medicine and Dentistry, University of Lancashire, Preston PR1 2HE, UK

**Keywords:** hepatocellular carcinoma, magnetic resonance imaging, nnU-Net, tumour segmentation, external validation, domain shift, multiphasic MRI, false positives, computed tomography

## Abstract

Background/Objectives: To establish an auditable nnU-Net v2 HCC MRI segmentation benchmark, quantify phase- and cohort-dependent transportability without external fine-tuning, evaluate false-positive safety on HCC-negative examinations, and test whether complementary arterial-plus-venous input improves performance in OpenSwissHCC. Methods: A 3D full-resolution nnU-Net was trained by five-fold cross-validation on 60 public ATLAS cases. The unchanged five-fold ensemble was applied separately to arterial, portal/venous, delayed and native T1-weighted images in LiverHccSeg and OpenSwissHCC. All 132 OpenSwissHCC subjects (63 HCC-positive and 69 HCC-negative) underwent inference in every phase; positive-case overlap and detection were reported separately from negative-examination specificity. A patient-paired five-fold ablation compared venous-only with arterial-plus-venous OpenSwissHCC training. MRI-to-CT application to 97 valid-reference HCC-TACE-Seg cases was exploratory. Results: The ATLAS out-of-fold mean Dice was 0.5315 (95% CI 0.4478–0.6126). The OpenSwissHCC positive mean Dice was 0.3388 arterial, 0.1267 venous, 0.1810 delayed and 0.0814 native; the corresponding detection was 55.6%, 28.1%, 27.6% and 23.8%, while the negative-examination specificity ranged from 50.7% to 81.2%. The detection was lower when all of the documented HCCs were <20 mm. Arterial-plus-venous training increased the mean Dice from 0.2269 to 0.3705 (paired difference +0.1435, 95% CI +0.0520 to +0.2343; *p* = 0.00223) and reduced the false-positive voxel burden (*p* = 0.0394), although the binary detection increase was not significant by exact McNemar testing (*p* = 0.0639). The MRI-to-CT mean Dice was 0.0286. Conclusions: The HCC segmentation performance was strongly domain-sensitive. Multiphasic input improved segmentation quality within the cohort, but reliable clinical use requires substantially broader development and prospective validation.

## Simple Summary

Artificial intelligence may help delineate hepatocellular carcinoma (HCC) on liver MRI, but apparently good performance within one dataset may deteriorate when scanner, contrast phase, lesion spectrum, annotation practice or clinical population changes. We established a reproducible five-fold nnU-Net benchmark on the public ATLAS MRI cohort, then applied the unchanged ensemble to independent MRI cohorts one phase at a time as a controlled domain-shift stress test. We also evaluated all HCC-negative OpenSwissHCC examinations to quantify false-positive safety and added a separate within-cohort experiment comparing venous-only with arterial-plus-venous training. External performance remained cohort- and phase-dependent; small-tumour subjects were harder to detect, and the false-positive burden was substantial. The arterial-plus-venous input improved segmentation quality compared with the venous-only training, but did not eliminate missed tumours or false positives. Direct MRI-to-CT transfer was near-nonfunctional. These findings support larger, multiphasic, multi-centre and lesion-aware development, rather than immediate clinical deployment.

## 1. Introduction

Hepatocellular carcinoma (HCC) is the most common primary liver malignancy and is frequently diagnosed, staged and monitored using multiphasic contrast-enhanced CT or MRI [1,2]. Three-dimensional tumour delineation can support tumour-burden quantification, treatment planning, response assessment, radiomics and longitudinal imaging biomarkers. Manual segmentation is time-consuming and vulnerable to variability related to lesion size, multiplicity, enhancement pattern, infiltrative growth and cirrhotic background liver.

Deep convolutional networks, particularly U-Net-derived architectures, dominate medical image segmentation [3]. nnU-Net is a strong reference framework because it self-configures preprocessing, network topology and training for a specified dataset [4]. A favourable internal Dice score does not, however, establish transportability. Liver MRI datasets differ in contrast phase, sequence, scanner, spatial resolution, lesion spectrum, annotation philosophy and target-class definition.

Published HCC MRI segmentation studies have reported tumour Dice values of approximately 0.68–0.92 using multiphasic U-Net, three-dimensional convolutional, recurrent or self-configuring pipelines [5,6,7,8,9,10]. These estimates arise from different case partitions, phase combinations, target definitions, post-processing rules and aggregation strategies. Direct leaderboard interpretation is, therefore, unsafe, and independent testing should be accompanied by explicit characterisation of domain shifts.

Routine HCC interpretation is multiparametric: radiologists integrate precontrast, arterial, portal/venous, delayed and often T2-weighted or diffusion-sensitive information. Consequently, isolated-phase inference is not a surrogate for clinical radiological interpretation. In this study, phase-wise external inference is deliberately used as a controlled single-phase domain-shift stress test. A separate patient-paired within-OpenSwissHCC ablation then asks a different question: whether adding aligned arterial information to venous input improves segmentation when training and testing remain within the same cohort.

Case-level Dice alone also obscures failure mechanisms. A score of zero may represent complete lesion non-detection, whereas detected-only Dice can appear favourable despite many missed lesions. Lesion precision, recall, F1, negative-examination specificity, false-positive burden, volume error and surface-distance measures, therefore, provide complementary information [11,12]. The reporting was guided by CLAIM, where applicable [13].

**Figure 1:**
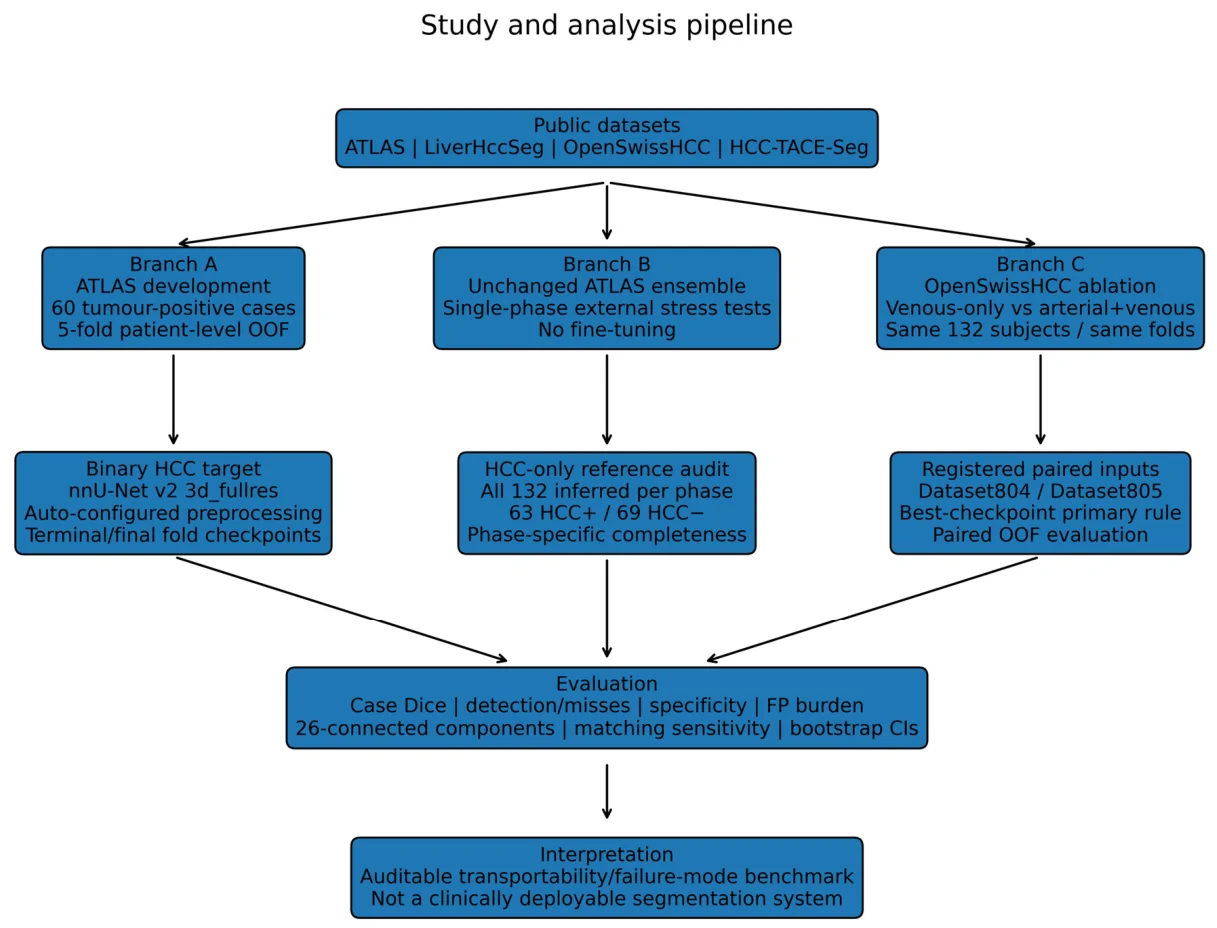
The processing pipeline, showing the distinct ATLAS development/internal OOF branch, unchanged-model external single-phase stress-test branch, and within-OpenSwissHCC multiphasic ablation. The branches are analytically separate and are not pooled into a single validation claim. The public source datasets are cited in References [14,15,16,17].

The study objectives were to: (1) establish a completed five-fold ATLAS benchmark; (2) quantify cross-sectional ATLAS phase-stratum performance; (3) apply the unchanged ATLAS ensemble to independent MRI cohorts one phase at a time, without external tuning; (4) process the full 132-subject OpenSwissHCC cohort, including all 69 HCC-negative examinations; (5) assess matching-rule sensitivity and lesion-size/multiplicity effects; (6) compare all paired external phases in complete cases; (7) perform a locked venous-only versus arterial-plus-venous within-cohort ablation; and (8) retain MRI-to-CT transfer only as an exploratory extreme-domain-shift stress test. The complete analysis flow is summarised in Figure 1.

## 2. Materials and Methods

### 2.1. Study Design, Data Provenance and Ethics

This retrospective methodological study used only de-identified public imaging datasets and released annotations/metadata. No local clinical images were included. Dataset801_HCCMRI_ATLAS is an nnU-Net-format repackaging of the public ATLAS training set, not a private institutional cohort. ATLAS was used for development and internal out-of-fold (OOF) benchmarking; LiverHccSeg and OpenSwissHCC were used for independent MRI domain-shift evaluation; OpenSwissHCC also supported a separate within-cohort multiphasic ablation; and HCC-TACE-Seg was retained only for exploratory MRI-to-CT testing. Table 1 defines all cohort roles.

### 2.2. ATLAS Development Cohort and Tumour-Mask Preparation

The development cohort comprised the 60 training cases released with the ATLAS challenge [14]. Each case contained one liver-focused T1-weighted MRI volume and a label map with background, liver and tumour classes. For binary tumour segmentation, the tumour was mapped to foreground label 1 and the liver/background to 0. No additional manual contour editing, smoothing or case exclusion was performed. The released metadata comprised 33 arterial-phase, 10 portal-venous, 8 delayed, 7 phase-unknown and 2 non-contrast examinations. Because each ATLAS participant contributes only one selected volume, these phase categories are cross-sectional patient/covariate strata rather than repeated phases within the same patient; they cannot support within-patient causal inference about contrast phase.

The released reference masks were used as supplied and were not independently re-adjudicated. The source publication describes systematic liver/tumour annotation for treatment-planning research by a multidisciplinary radiology and nuclear-medicine team [14]. The imaging contours represent radiologically visible tumour extent rather than an absolute histopathological biological boundary.

### 2.3. nnU-Net Configuration, Training and Internal Validation

Training used nnU-Net v2.6.4 with the standard nnUNetTrainer and 3d_fullres configuration [4]. No manual grid search, Bayesian optimisation, or external-performance-driven hyperparameter tuning was performed. The archived nnUNetPlans and trainer configuration are summarised in Table 2. All five folds used 48 training and 12 validation patients. The internal OOF benchmark used the terminal/final checkpoint from each completed fold, consistent with the archived training/validation runs; no retrospective substitution of best checkpoints was performed. The same five terminal/final-fold models formed the unchanged external ensemble. Standard nnU-Net raw and post-processed OOF validation summaries were numerically identical. No manually selected probability threshold, tumour-size filter, connected-component removal, contour smoothing, or other retrospective tumour-specific post-processing was applied.

The internal performance was calculated from the combined OOF predictions for all 60 cases. The primary overlap statistic was the mean of the 60 patient-level tumour Dice values, which weights patients equally. For direct reconciliation with the published ATLAS comparator, a dataset-aggregated Dice was also calculated by pooling tumour true-positive, false-positive and false-negative voxels across all 60 OOF predictions before computing the Dice. As a post hoc domain-characterisation analysis, OOF predictions were stratified by released ATLAS phase metadata; this stratification is descriptive and cross-sectional.

### 2.4. External MRI Cohorts and Reference Construction

#### 2.4.1. LiverHccSeg

LiverHccSeg is a public multiphasic MRI dataset with manual liver and HCC segmentations from two board-certified abdominal radiologists [15]. Seventeen examinations were available, of which 14 contained non-empty HCC references. The primary tumour-positive analyses, therefore, used the same 14 cases across arterial, portal-venous, delayed and native T1-weighted phases, with a consistent rater-1 tumour reference. Tumour-empty cases were retained only in detailed outputs and did not inflate tumour-positive Dice.

#### 2.4.2. OpenSwissHCC

OpenSwissHCC contains 132 subjects: 63 HCC-positive and 69 HCC-negative examinations, with lesion-level diagnostic labels and manual segmentations [16]. The metadata contain 97 lesions labelled HCC. Phase-specific HCC references were reconstructed by selecting only lesions labelled HCC = 1 and unioning the corresponding phase-specific masks; non-HCC lesions remained in the background. The unchanged ATLAS ensemble was run on all 132 subjects in every T1 phase, independent of prior prediction availability. HCC-positive reference completeness was arterial 63/63, venous 57/63, delayed 58/63 and native 42/63. Reduced positive denominators, therefore, reflect incomplete released manual references, not inference selection. All 69 HCC-negative examinations were included for specificity and false-positive analyses.

### 2.5. External Single-Phase Inference as a Domain-Shift Stress Test

The unchanged ATLAS five-fold ensemble was applied separately to arterial, portal-venous/venous, delayed and native T1-weighted inputs. No external fine-tuning, intensity harmonisation beyond nnU-Net preprocessing, threshold selection or external-label-driven checkpoint selection was performed. These experiments are intentionally artificial single-phase stress tests of domain sensitivity; they do not emulate routine multiparametric HCC interpretation.

### 2.6. Within-OpenSwissHCC Multiphasic Ablation

To test complementary phase information without conflating it with external validation, two new nnU-Net datasets were created from the same 132 registered OpenSwissHCC subjects and the same fixed five patient-level splits. Dataset804 used venous input only; Dataset805 used aligned arterial plus venous channels. Both used the same frozen HCC-only master labels (63 positive and 69 negative). The primary checkpoint rule for these ablations was checkpoint_best, selected automatically by nnU-Net EMA foreground pseudo-Dice; the final checkpoints were not retrospectively chosen to improve reported outcomes. The Dataset804/805 results are, therefore, within-cohort development/ablation results and do not replace the unchanged-ATLAS-model external evaluation.

### 2.7. Exploratory MRI-to-CT Stress Test

HCC-TACE-Seg contains pre- and post-procedural multiphasic CT imaging with released tumour segmentation [17]. The primary analysis used 97 pre-procedural portal-venous CT cases with valid non-empty tumour references. Seven additional extracted masks were technically unusable because DICOM slice–coordinate mismatch yielded empty references and were excluded. The ATLAS MRI ensemble was applied directly without retraining, harmonisation or domain adaptation. Because CT masks were frequently fragmented, the lesion-wise results were interpreted as connected-component performance, rather than clinical tumour counts.

### 2.8. Metrics, Matching and Failure Classification

Binary positive-case Dice was the primary overlap metric. For HCC-negative examinations, true-negative scans were not assigned Dice = 1 and pooled with positive cases; instead, specificity, false-positive scan rate, false-positive component count and false-positive volume were reported separately. Additional outcomes included any-overlap case detection, voxel sensitivity, tumour-volume error, 95th-percentile Hausdorff distance (HD95) and average symmetric surface distance (ASSD), where valid.

The reference and predicted masks were decomposed using 3D 26-neighbour connectivity. The primary component analysis used one-to-one matching when any voxel overlap occurred. Matching sensitivity compared greedy assignment with maximum-score Hungarian assignment and tested any-overlap plus IoU thresholds of 0.01, 0.05 and 0.10. Matched-lesion Dice was explicitly conditional on successful spatial localisation and was not interpreted as overall performance. Complete detection failure denotes a non-empty reference with no overlap.

### 2.9. Statistical Analysis

The mean case Dice was accompanied by non-parametric 95% confidence intervals from 100,000 case-bootstrap resamples with a fixed seed [18]. Binomial detection/specificity intervals were calculated for safety summaries. Internal ATLAS phase groups were compared exploratorily using Kruskal–Wallis testing. External paired phase analyses used subjects with complete measurements in all four phases: 14 tumour-positive LiverHccSeg subjects and 38 OpenSwissHCC subjects with complete phase-specific HCC references. Within each cohort, a four-phase Friedman test was followed, when significant, by all six paired Wilcoxon signed-rank contrasts with Holm correction; paired mean Dice differences were accompanied by case-bootstrap confidence intervals. The OpenSwissHCC lesion-size/burden analysis compared subjects with all documented HCCs < 20 mm against subjects with at least one HCC ≥ 20 mm. A pooled cross-cohort phase-by-size causal model was not used because ATLAS phase categories comprise different participants, whereas LiverHccSeg and OpenSwissHCC provide repeated phases within participants. The Dataset804/805 ablation used paired Wilcoxon tests for continuous paired outcomes and exact McNemar tests for binary detection/specificity. No statistical comparison was used to select an external model or threshold.

## 3. Results

### 3.1. Internal Five-Fold ATLAS Benchmark

**Figure 2:**
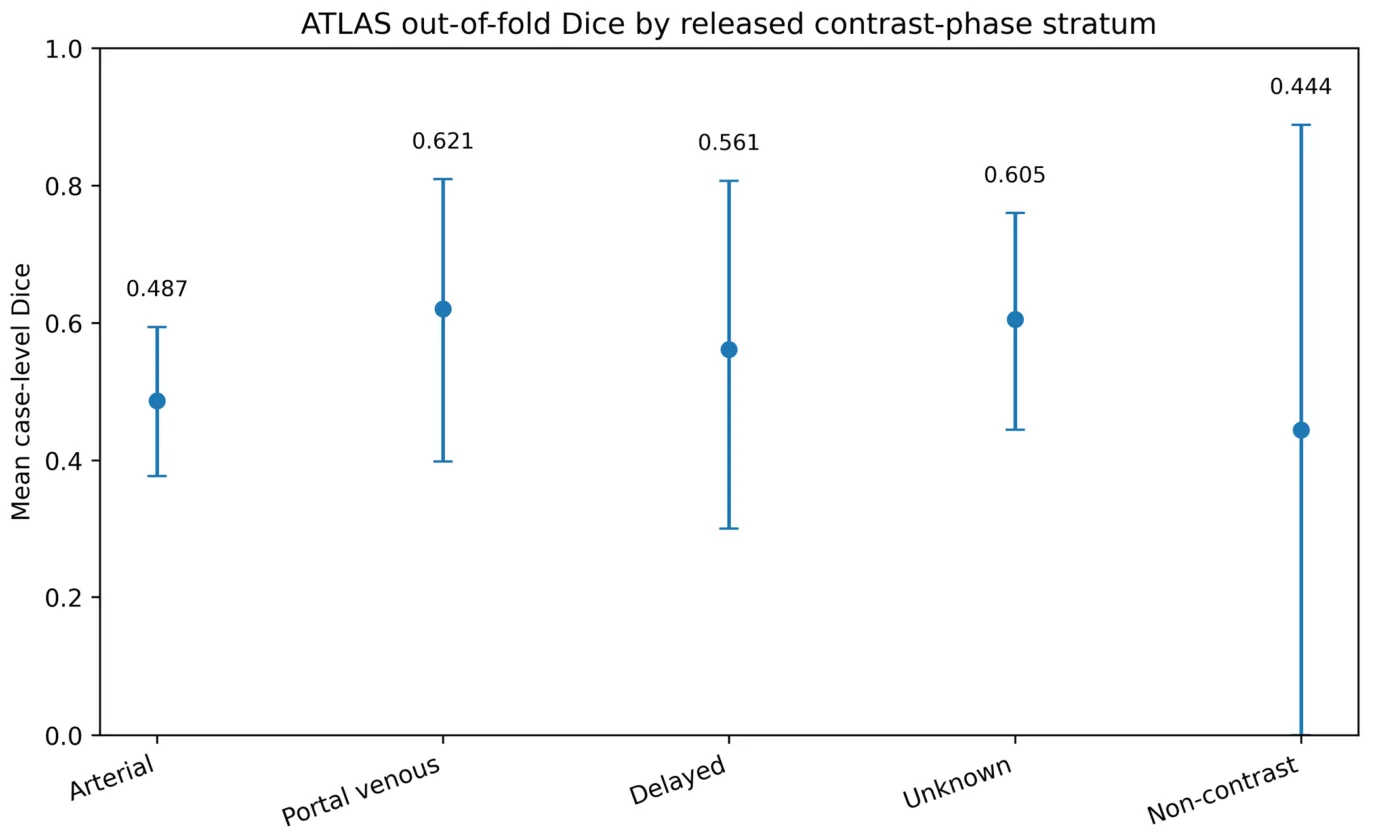
ATLAS internal OOF mean Dice stratified by released contrast-phase metadata. Error bars are case-bootstrap 95% confidence intervals. Phase groups contain different participants and are cross-sectional covariate strata, not repeated measurements.

All five ATLAS folds were completed. Across 60 combined OOF predictions, the mean per-case Dice was 0.5315 (bootstrap 95% CI 0.4478–0.6126), the median Dice was 0.6317, and the IQR was 0.2491–0.8303; the fold means ranged from 0.4112 to 0.6784. Pooling tumour TP, FP and FN voxels across the same 60 OOF predictions yielded a dataset-aggregated Dice of 0.6231 (aggregate IoU 0.4526, voxel precision 0.8043 and voxel recall 0.5086). Phase-stratified performance is reported in Table 3 and Figure 2. The exploratory Kruskal–Wallis comparison was not significant (*p* = 0.748), but small non-arterial strata produced wide confidence intervals and did not establish phase equivalence.

### 3.2. LiverHccSeg Single-Phase External Stress Test

**Figure 3:**
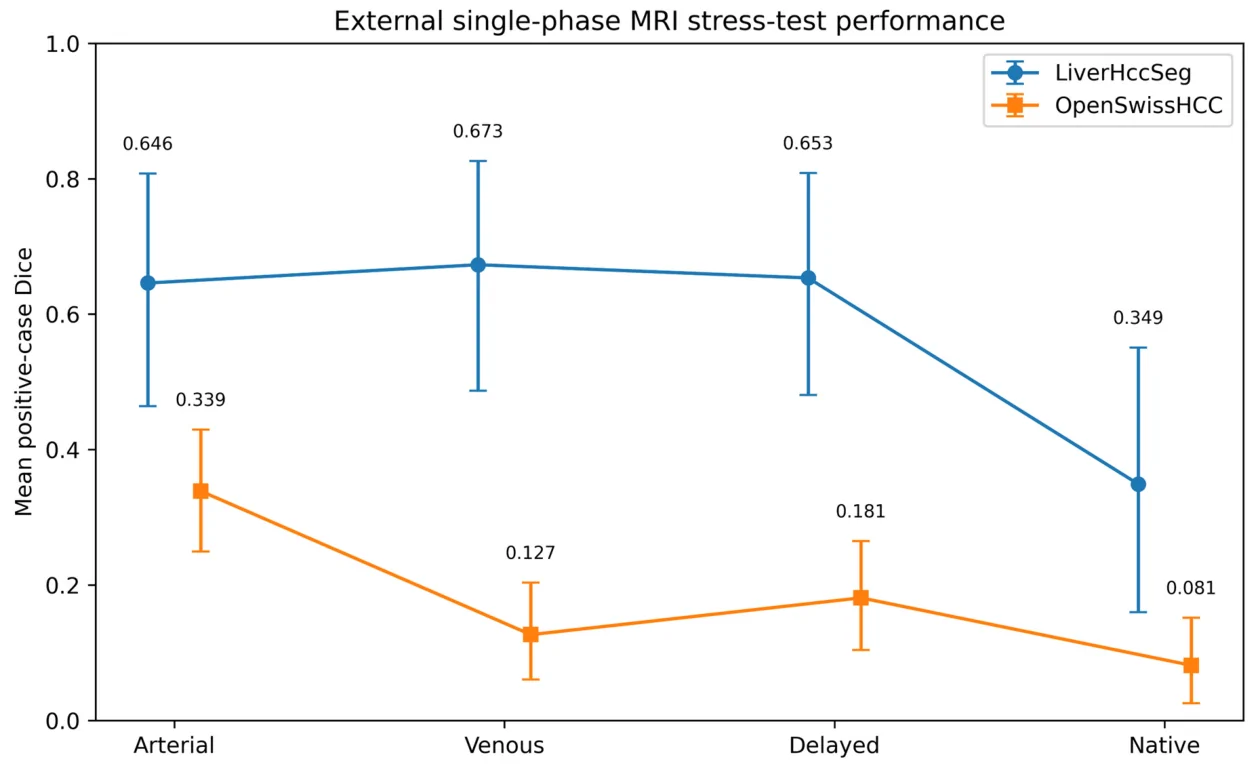
The external single-phase MRI stress-test mean positive-case Dice, with case-bootstrap 95% confidence intervals. OpenSwissHCC uses the full-inference cohort with phase-specific positive-reference completeness; LiverHccSeg uses the same 14 tumour-positive cases in all phases.

In the same 14 tumour-positive LiverHccSeg cases, the mean Dice was 0.6460 (95% CI 0.4640–0.8077) for arterial, 0.6730 (0.4871–0.8262) for portal-venous, 0.6535 (0.4806–0.8087) for delayed and 0.3490 (0.1600–0.5507) for native. Any-overlap case detection was 12/14, 12/14, 14/14 and 8/14, respectively. These results are included in OpenSwissHCC in Table 4 and Figure 3; the harmonised paired four-phase inferential analysis is reported in Section 3.6.

### 3.3. Full-Cohort OpenSwissHCC External Stress Test and Negative-Case Safety

All 132 OpenSwissHCC subjects underwent inference in every phase. Among HCC-positive subjects with complete phase-specific references, arterial imaging had the highest observed mean Dice (0.3388) and any-overlap case detection (55.6%), but 28/63 arterial positive cases remained complete misses. The venous, delayed and native positive-case mean Dice were 0.1267, 0.1810 and 0.0814, respectively (Table 4; Figure 3).

**Figure 4:**
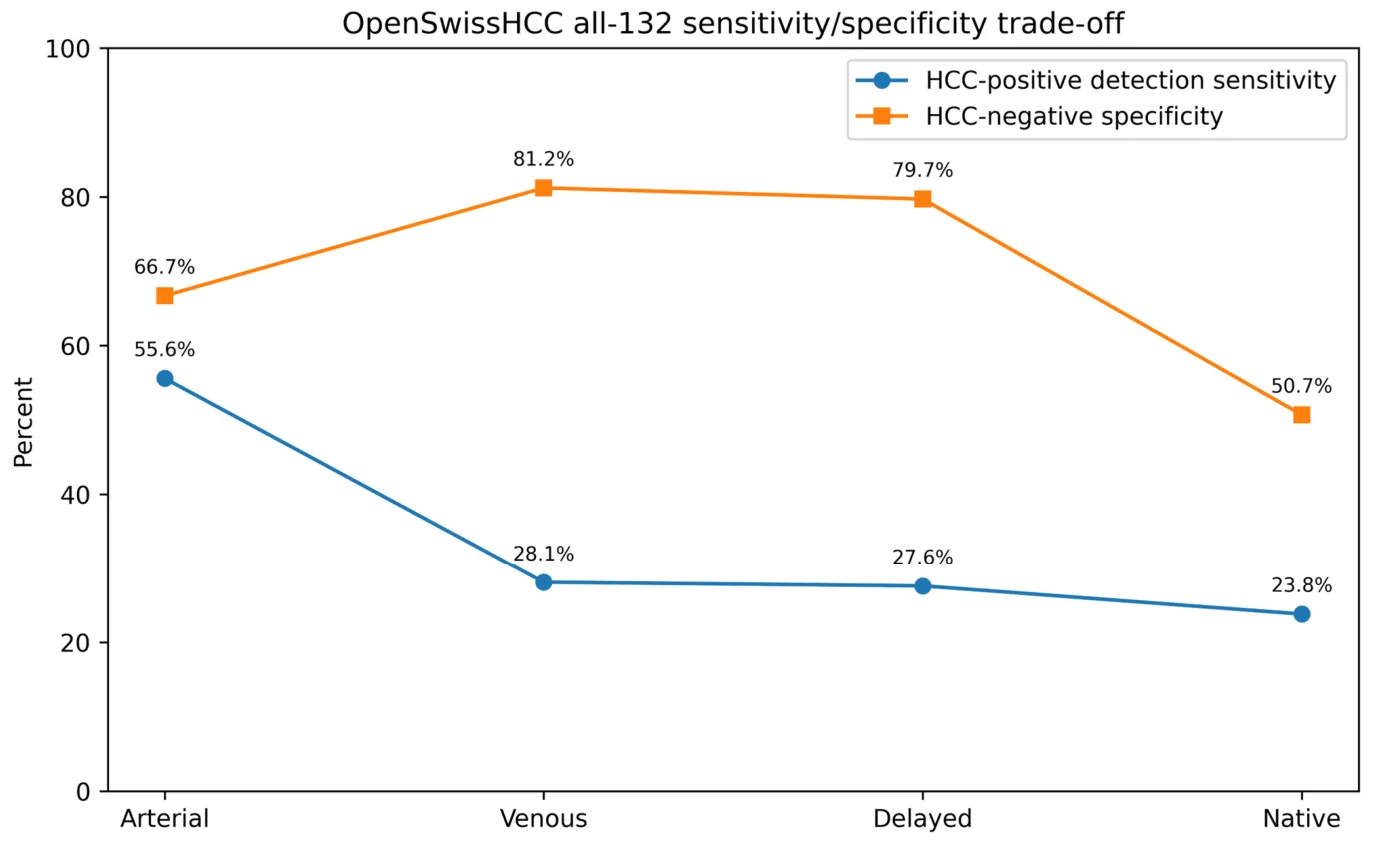
OpenSwissHCC all-132 case-level positive detection sensitivity and HCC-negative specificity by phase. No universal clinical-utility threshold is superimposed because no validated single technical cut-off establishes clinical readiness for HCC segmentation.

The 69 HCC-negative OpenSwissHCC examinations exposed a phase-dependent sensitivity/specificity trade-off. Specificity was 66.7% arterial, 81.2% venous, 79.7% delayed and 50.7% native (Table 5; Figure 4). Arterial produced false-positive foreground on 23/69 negative scans and native on 34/69. These negative cases were not pooled into an overall Dice estimate.

### 3.4. Matching-Rule Sensitivity and Conditional Lesion Metrics

The frozen component-level sensitivity analysis used complete-reference component sets (arterial n = 59, venous n = 57, delayed n = 56, and native n = 39) to isolate the effect of lesion-matching rules. Greedy and Hungarian one-to-one assignments produced identical TP/FP/FN counts at every tested threshold. Increasing the criterion from any overlap to IoU ≥ 0.10 reduced arterial lesion recall from 42.7% to 39.3%, venous from 21.2% to 14.1%, delayed from 20.7% to 19.5% and native from 16.4% to 9.1% (Table 6). The qualitative finding of substantial external detection failure was unchanged.

### 3.5. Lesion-Size, Tumour-Burden and Multiplicity Spectrum

**Figure 5:**
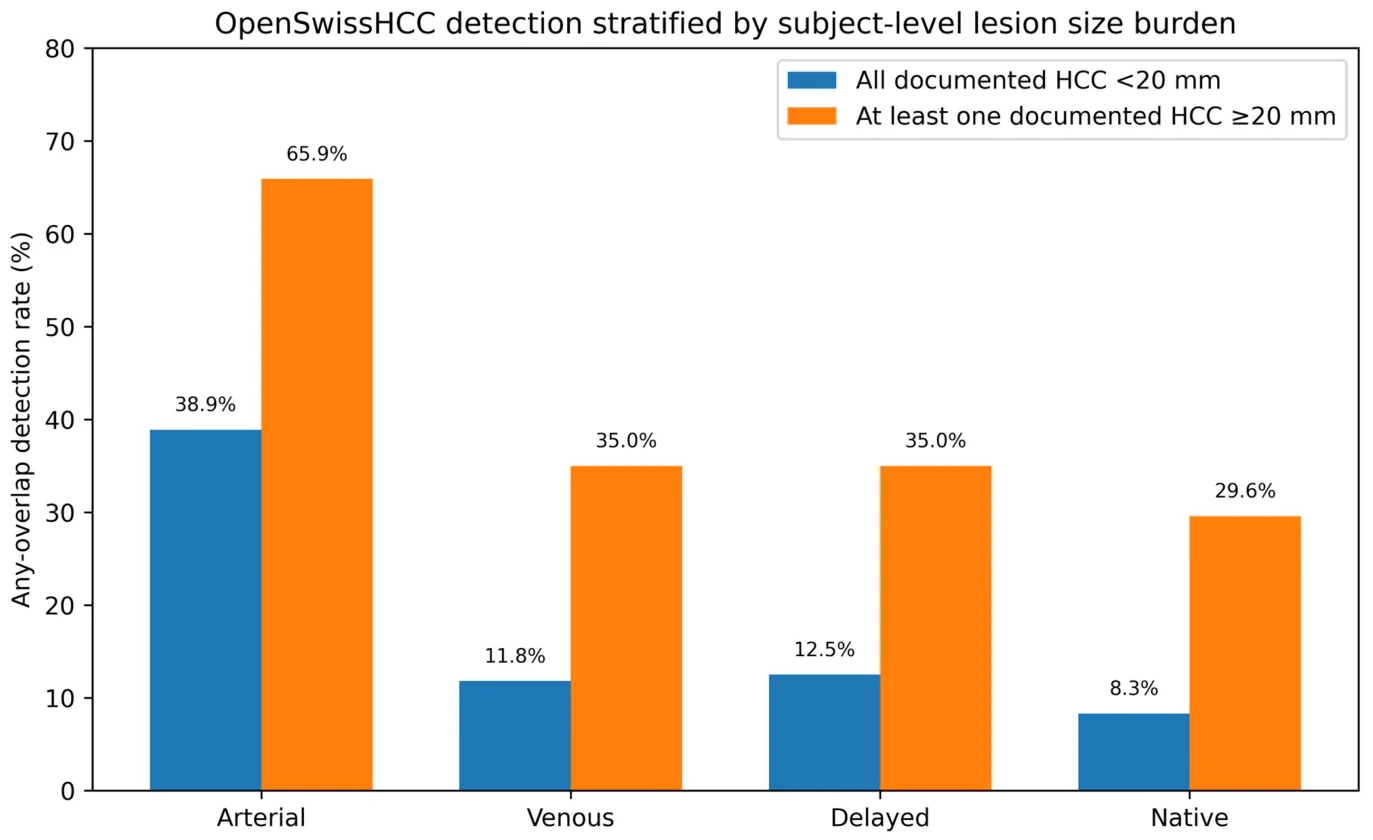
OpenSwissHCC any-overlap case detection, stratified by the subject-level lesion-size burden. The analysis is descriptive and does not disentangle lesion size from other cohort-spectrum differences.

Subject-level OpenSwissHCC lesion-size/burden stratification showed consistently lower detection when all documented HCCs were <20 mm. Arterial detection was 38.9% in the all-small stratum versus 65.9% when at least one HCC was ≥20 mm; the same direction occurred in every phase (Table 7; Figure 5). The mean Dice was likewise lower in the all-small stratum. Multifocal cases did not show worse crude detection; this is not interpreted as a beneficial effect of multifocality because tumour burden and the number of opportunities for overlap are confounded.

The three MRI datasets do not support a single pooled causal phase-by-size analysis because their sampling structures differ: ATLAS contributes one selected T1-weighted phase per participant, whereas LiverHccSeg and OpenSwissHCC provide repeated phases within the same subjects. Importantly, the formal phase comparisons in LiverHccSeg and in the complete four-phase OpenSwissHCC subset are patient-paired, so each subject’s lesion-size spectrum and tumour burden are held constant across the phase contrast. The separate Dataset804/805 ablation likewise uses identical OpenSwissHCC subjects, labels, and patient splits. These designs, therefore, show that lesion size is an important co-driver of difficulty but cannot by itself explain the observed within-subject phase/input effects.

### 3.6. Complete Four-Phase Paired Comparisons

In LiverHccSeg, all 14 tumour-positive subjects had complete arterial, portal-venous, delayed and native measurements. The Friedman test was significant (chi-square = 8.7985, *p* = 0.03209). All six paired Wilcoxon contrasts were evaluated with Holm correction. Only arterial versus native remained significant (mean arterial-minus-native Dice difference +0.2971, bootstrap 95% CI +0.1305 to +0.4772; Holm-adjusted *p* = 0.0459). Arterial versus portal-venous (difference −0.0270; Holm *p* = 1.000), arterial versus delayed (−0.0075; *p* = 1.000), portal-venous versus delayed (+0.0195; *p* = 1.000), portal-venous versus native (+0.3241; *p* = 0.1145) and delayed versus native (+0.3046; *p* = 0.1675) were not significant after multiplicity correction.

**Figure 6:**
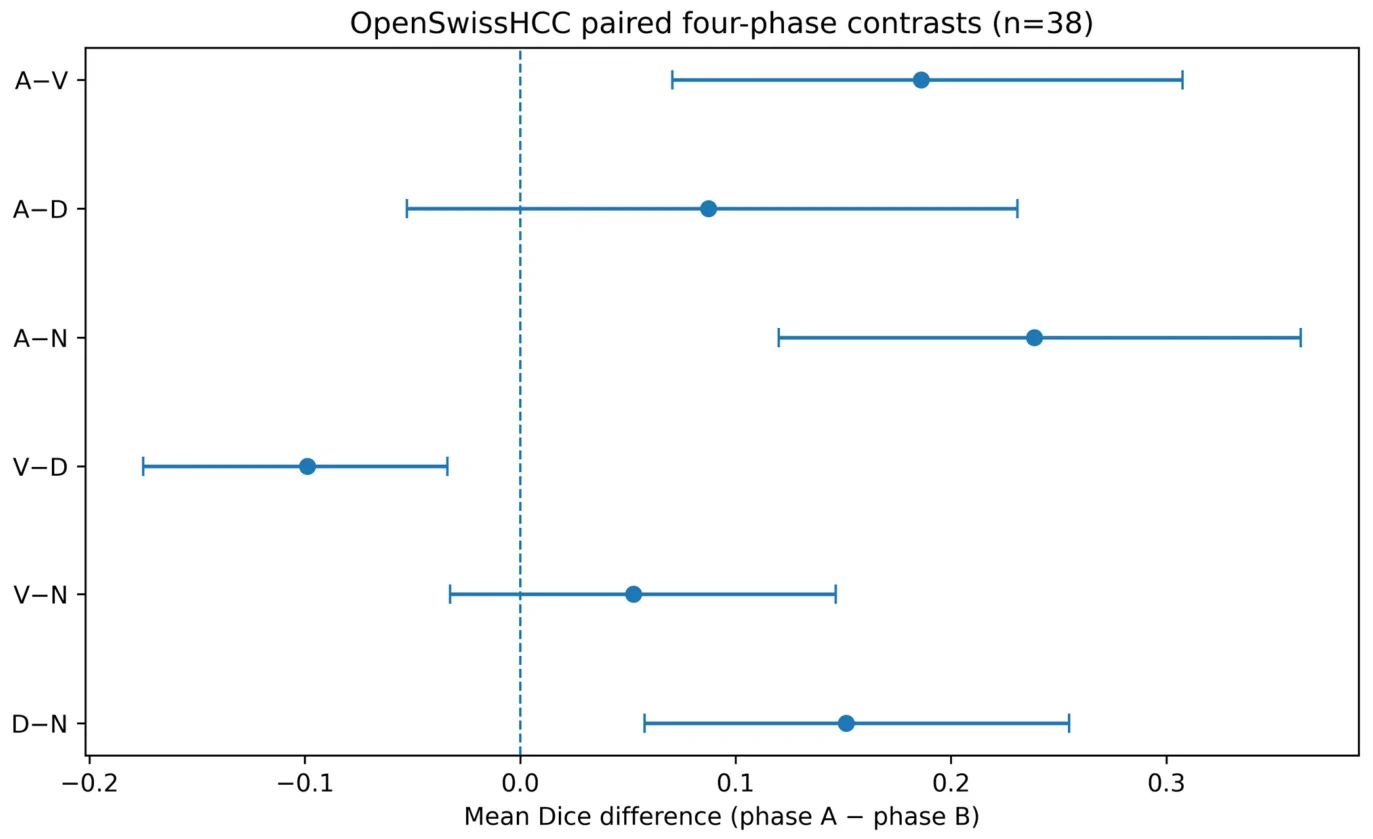
The paired mean Dice differences with bootstrap 95% confidence intervals in the 38 OpenSwissHCC subjects, with complete references in all four phases. A, arterial; V, venous; D, delayed; N, native.

Among 38 OpenSwissHCC subjects with complete references in all four phases, the Friedman test was significant (chi-square = 8.3134, *p* = 0.03996). All six pairwise Wilcoxon contrasts were then evaluated with Holm correction (Table 8; Figure 6). Arterial exceeded venous (Holm *p* = 0.0382) and native (*p* = 0.0141); delayed exceeded venous (*p* = 0.0382) and native (*p* = 0.0388). Arterial versus delayed and venous versus native were not significant. These paired results supersede selective emphasis on a single arterial-versus-venous contrast.

### 3.7. Within-OpenSwissHCC Venous-Only Versus Arterial-Plus-Venous Ablation

**Figure 7:**
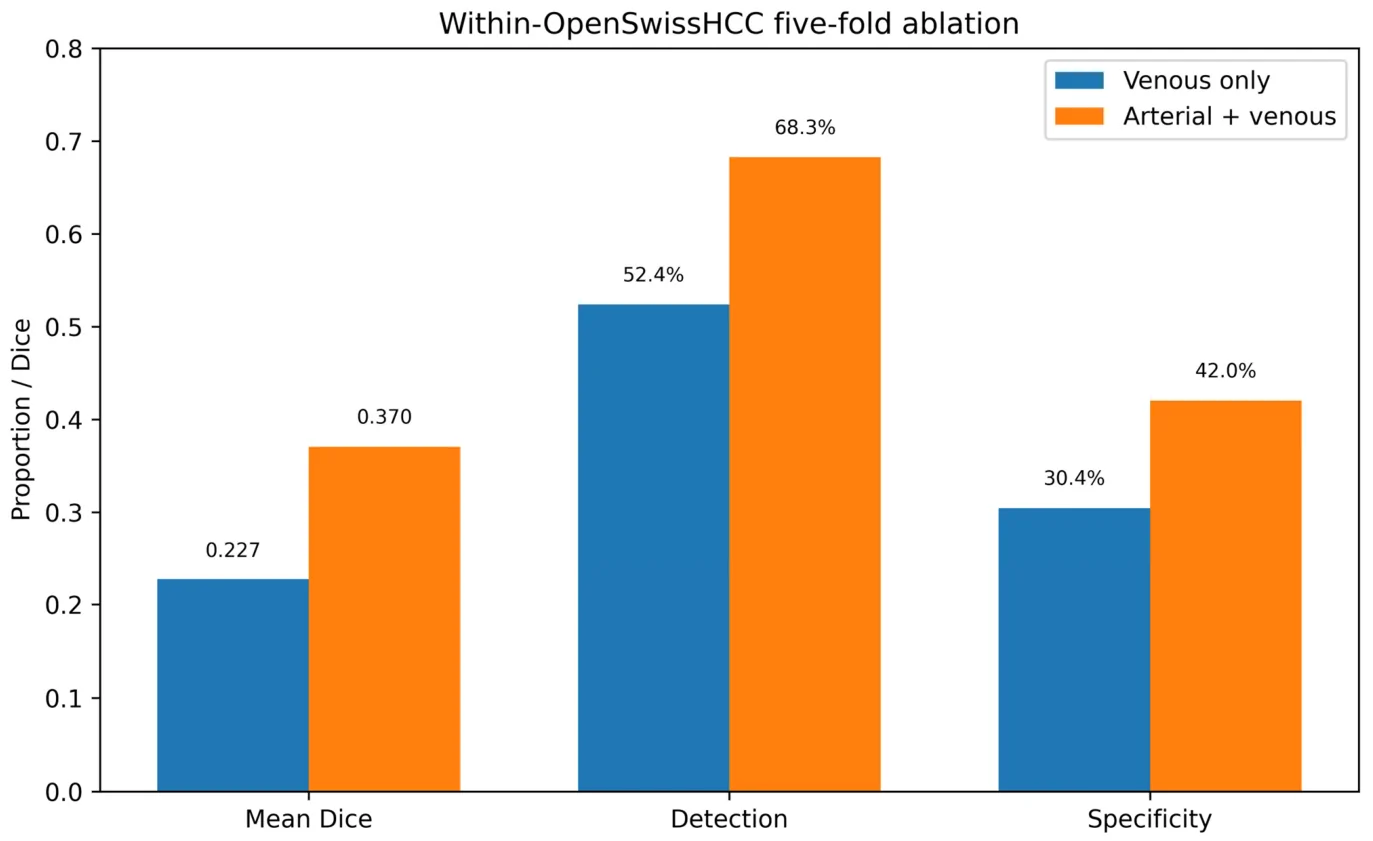
Within-OpenSwissHCC five-fold ablation comparing the venous-only with aligned arterial-plus-venous input using identical patient splits. The plot shows the mean Dice, any-overlap detection and correct-empty specificity; formal paired tests are reported in Table 9.

Using identical patient splits and frozen HCC-only labels, arterial-plus-venous training improved the HCC-positive mean Dice from 0.2269 to 0.3705 (paired difference +0.1435, 95% CI +0.0520 to +0.2343; Wilcoxon *p* = 0.00223) and the median Dice from 0.0249 to 0.4245. The mean per-case voxel sensitivity improved (*p* = 0.00485). Any-overlap detection increased from 52.4% to 68.3%, but the exact McNemar *p*-value was 0.0639 and is, therefore, described as a numerical, not statistically significant, binary-detection increase. On 69 HCC-negative scans, the mean false-positive voxel burden decreased from 640.3 to 209.8 voxels per negative scan (paired *p* = 0.0394); specificity increased numerically from 30.4% to 42.0% (McNemar *p* = 0.2005). Table 9 and Figure 7 summarise the primary paired results.

### 3.8. Exploratory MRI-to-CT Stress Test

**Figure 8:**
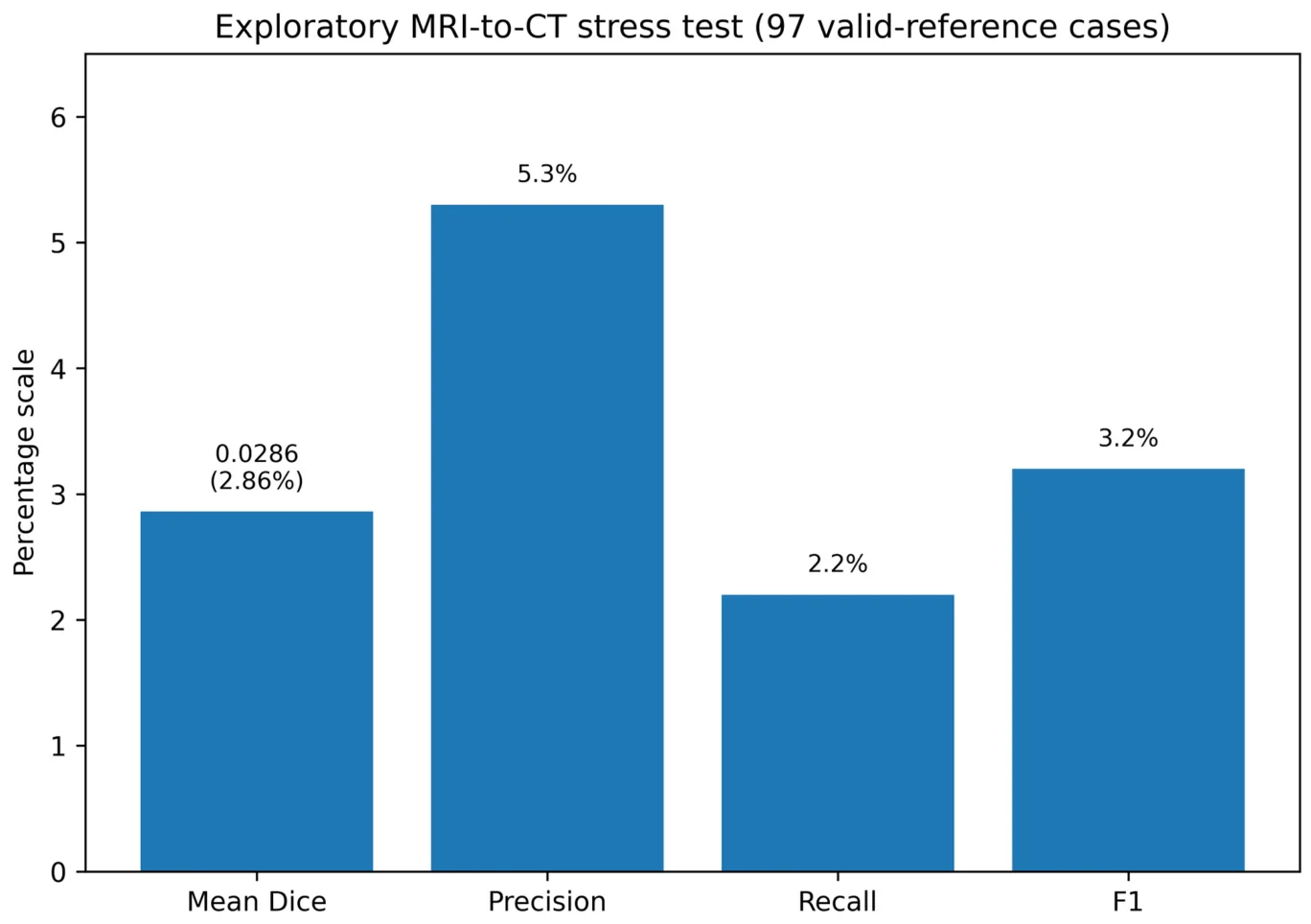
Exploratory MRI-to-CT stress-test summary for 97 valid-reference HCC-TACE-Seg cases. Mean Dice is displayed on a percentage scale (0.0286 = 2.86%) only for visual comparability with component precision, recall and F1.

Direct application of the MRI-trained ensemble to 97 HCC-TACE-Seg cases with valid pre-procedural portal-venous CT tumour references yielded a mean Dice of 0.0286 (bootstrap 95% CI 0.0100–0.0522) and a median Dice of 0. The component analysis contained 847 reference components, 357 predicted components, and 19 matches, giving precision 5.3%, recall 2.2%, F1 3.2%, and 3.48 false-positive components per case (Table 10; Figure 8). This experiment demonstrates extreme modality mismatch and is not CT validation.

### 3.9. Contextual Comparison and ATLAS Protocol Reconciliation

The selected published HCC MRI segmentation studies are summarised in Table 11. The cross-study values are not a leaderboard because cohorts, phase combinations, target definitions, partitions, post-processing and aggregation differ. Karabağ et al. [10] report a tumour Dice of 0.915 +/− 0.016 for five-fold validation and 0.892 for a separate held-out test set; the latter is not their five-fold result. The present tumour-only task maps liver/background to 0 and uses the unmodified released tumour masks. The mean per-case OOF Dice is 0.5315, while the dataset-aggregated pooled-voxel Dice across the same 60 OOF masks is 0.6231. Relative to the Karabağ five-fold estimate of 0.915, the absolute gaps are, therefore, 0.3835 (per-case) and 0.2919 (pooled-voxel); relative to their held-out 0.892, the corresponding gaps are 0.3605 and 0.2689. Standard nnU-Net post-processing produced no numerical change in our OOF summary, and no manual tumour-specific filtering or threshold optimisation was applied. Aggregation, therefore, explains only part of the discrepancy. The differences in task construction, case partitioning, nnU-Net version/plans, checkpoint policy, aggregation and incompletely reported post-processing may contribute; however, the published comparator does not provide sufficient fold-level and implementation detail for an unambiguous identical reconstruction. The residual difference cannot be fully explained, and no direct leaderboard equivalence is claimed (Table 12).

## 4. Discussion

### 4.1. Principal Findings

This study provides an audited reference benchmark rather than a new architecture. Four findings are most important. First, the 60-case ATLAS five-fold OOF baseline achieved moderate overlap with substantial fold-to-fold uncertainty. Second, unchanged-model external MRI performance was strongly cohort- and phase-dependent, with frequent complete misses and substantial false-positive burden. Third, the lesion spectrum mattered: the subjects with only sub-20 mm HCCs were consistently harder to detect. Fourth, adding aligned arterial information to the venous input improved the segmentation quality and reduced false-positive voxel burden within OpenSwissHCC, but did not eliminate missed lesions or yield a statistically significant binary-detection increase.

### 4.2. Phase, Cohort and Domain Shift

The single-phase external experiment is intentionally narrower than clinical multiparametric HCC assessment. It shows that a fixed segmentation model is sensitive to contrast timing and cohort context, not that one phase should replace multiparametric interpretation. In LiverHccSeg, the Friedman test was significant, but after Holm correction, only arterial versus native remained significant; the three post-contrast phases did not differ significantly from one another. In OpenSwissHCC, paired four-phase testing confirmed significant contrasts for arterial versus venous/native and delayed versus venous/native, while arterial versus delayed and venous versus native were not significant. Because these external phase comparisons are within-subject, between-phase differences in patient-level tumour burden cannot account for the paired effects. The phase is, therefore, one contributor to domain shift, not a complete explanation.

Standard nnU-Net resampling, normalisation and augmentation reduce some low-level variation, but do not guarantee domain invariance. The observed degradation is direct evidence that scanner/vendor, field strength, sequence implementation, contrast timing, lesion spectrum, annotation structure and population differences remain relevant. Robust deployment would require multicentre/multivendor development, explicit acquisition diversity, separated calibration/adaptation and prospective site-level validation.

### 4.3. Lesion Discovery Versus Contour Quality

Detection failure is more clinically concerning than contour imperfection. The matching-sensitivity analysis showed that stricter IoU requirements reduced absolute lesion recall but did not change the overall conclusion of poor external discovery. The conditional matched-lesion Dice can, therefore, appear relatively favourable among successfully localised lesions and must not be interpreted as overall performance. This study deliberately leads interpretation with positive-case detection, complete misses, negative-case specificity and false-positive burden.

The size-burden analysis supports the lesion spectrum as an important co-driver. Detection and the mean Dice were consistently lower when all documented OpenSwissHCC HCCs were <20 mm. This analytical result supports, rather than merely speculates about, a small-lesion effect. However, lesion size does not fully explain the phase findings: LiverHccSeg and complete-case OpenSwissHCC phase tests compare the same patients across phases, and the Dataset804/805 ablation holds subjects, labels and folds constant. Multifocality did not show worse crude detection, probably because greater tumour burden creates more opportunities for overlap; this should not be interpreted causally. Future lesion-aware development should, therefore, emphasise small-lesion sampling, hard-negative mining and explicit burden stratification.

### 4.4. What the Multiphasic Ablation Adds

The paired Dataset804/805 ablation provides direct evidence that complementary phase information can improve segmentation quality when patient splits, labels and cohort are held constant. The mean Dice and voxel sensitivity improved significantly, and the negative-case false-positive voxel burden decreased. However, detection and specificity improvements were only numerical using exact McNemar testing. This distinction is important: better overlap among detected lesions does not by itself establish adequate case-level sensitivity or safety. The ablation is, therefore, supportive of multiphasic development, not evidence of clinical readiness and not a substitute for external multiphasic validation.

### 4.5. Clinical Interpretation and Teleradiology

An absolute biological HCC boundary is not directly observable on routine MRI. The public expert contours used here are imaging surrogates for radiologically visible tumour extent rather than histopathological whole-tumour boundaries. Future boundary research could combine multi-reader consensus, pathology correlation/co-registration, and, where feasible, uncertainty-aware modelling.

Potential teleradiology benefits of a mature segmentation system include asynchronous pre-segmentation, standardised tumour-burden quantification, prioritisation, second-reader support and longitudinal measurement. The present results also show the principal hazards: false reassurance from missed small HCCs, false-positive burden, sensitivity to acquisition protocol/phase, and workflow integration demands. No universal mean-Dice threshold defines clinical acceptability across detection, treatment planning, volumetry and longitudinal response. Clinical readiness is multidimensional and requires lesion sensitivity, acceptable false-positive burden, boundary/volume accuracy, calibrated uncertainty and prospective human–AI workflow evaluation. These data do not support autonomous teleradiology deployment.

### 4.6. Limitations

This study has several important limitations. First, the ATLAS development cohort contains only 60 subjects, resulting in 48 training and 12 validation patients per fold and substantial uncertainty in the internal benchmark. Second, ATLAS provides one selected T1-weighted volume per participant; its contrast-phase strata, therefore, represent different patients rather than repeated multiphasic observations and cannot support within-patient causal inference regarding phase. Third, the public datasets differ in scanner/vendor, field strength, acquisition protocol, contrast timing, spatial resolution, lesion spectrum and annotation structure. OpenSwissHCC has incomplete phase-specific positive HCC references, despite inference having been performed on all 132 subjects. Lesion size, total tumour burden, phase, and cohort spectrum remain partially confounded across datasets, and a single pooled cross-cohort phase-by-size causal coefficient would mix between-subject ATLAS contrasts with within-subject external contrasts. Reference contours were those released with the public datasets and were not independently re-adjudicated by additional abdominal radiologists; they represent imaging surrogates for radiologically visible tumour extent rather than absolute histopathological boundaries. Component-level metrics remain sensitive to matching definitions, although alternative matching algorithms and IoU thresholds did not materially change the conclusion of substantial external detection failure. The external single-phase experiments are deliberately artificial domain-shift stress tests and do not reproduce routine multiparametric HCC interpretation. The arterial-plus-venous experiment is a within-OpenSwissHCC development/ablation analysis, rather than independent external validation. Finally, the MRI-to-CT experiment is an extreme modality-shift stress test and has no direct deployment implication. These retrospective technical results do not establish clinical safety or utility; prospective multicentre, multivendor and clinically integrated validation is required before deployment.

### 4.7. Future Development

Future work should prioritise larger multicentre and multivendor cohorts with aligned multiphasic/multisequence imaging, lesion-aware sampling, small-HCC enrichment, hard-negative mining, false-positive control, calibrated detection thresholds, uncertainty estimation and prospective external testing. A strong development pathway would separate training, adaptation/calibration and final external sites, followed by reader studies or prospective human–AI workflow evaluation. The current results suggest that multiphasic input is a rational component of that pathway, but not sufficient by itself.

## 5. Conclusions

A standard 3D nnU-Net trained on the public 60-case ATLAS cohort provides a reproducible reference benchmark but does not generalise consistently across independent MRI cohorts. Single-phase external stress testing demonstrates marked phase and cohort sensitivity, while all-negative OpenSwissHCC evaluation exposes substantial false-positive safety limitations. Smaller-tumour subjects are harder to detect. A paired arterial-plus-venous ablation improves segmentation quality and reduces false-positive voxel burden compared with venous-only training but does not eliminate missed HCCs or establish clinical reliability. Direct MRI-to-CT transfer is near-nonfunctional. The appropriate next step is larger, multiphasic, lesion-aware, multicentre and prospectively validated development, rather than deployment of the present model.

## Figures and Tables

**Table 1:** Audited cohort definitions and evaluation roles.

Cohort	Input	Cases	Target Accounting	Role
ATLAS [14]	Single T1w MRI; 33 arterial, 10 portal-venous, 8 delayed, 7 phase-unknown, 2 non-contrasts	60	60 tumour-positive binary masks	Five-fold development/internal OOF benchmark
LiverHccSeg [15]	Arterial, portal-venous, delayed, native T1w MRI	17 total; 14 tumour-positive primaries	Consistent rater-1 HCC reference	Independent single-phase MRI stress test
OpenSwissHCC [16]	Arterial, venous, delayed, native T1w MRI; registered arterial + venous for ablation	132: 63 HCC+, 69 HCC−	97 documented HCC lesions; phase-specific HCC-only references; negatives all-zero	Independent single-phase stress test + separate paired within-cohort ablation
HCC-TACE-Seg [17]	Pre-procedural portal-venous CT	104 extracted; 97 valid-reference primaries	847 reference components in 97 valid non-empty tumour masks	Exploratory MRI-to-CT stress test

**Table 2:** The archived Dataset801 ATLAS nnU-Net v2.6.4 configuration used for the primary benchmark.

Parameter	Value
Configuration	nnUNetTrainer; nnUNetPlans; 3d_fullres
Input channels/labels	1 MRI channel; background = 0, tumour = 1
Target spacing	2.9999969 × 1.0416666 × 1.0416666 mm
Patch size/batch size	48 × 192 × 224 voxels/2
Normalisation	ZScoreNormalization; use_mask_for_norm = False
Image resampling	nnU-Net resample_data_or_seg_to_shape; data order = 3, z-order = 0
Segmentation resampling	Label-aware nnU-Net segmentation resampling; order = 1, z-order = 0
Architecture	PlainConvUNet; 6 stages; features 32,64,128,256,320,320
Kernels	[1,3,3] first stage; [3,3,3] thereafter
Strides	[1,1,1], [1,2,2], [2,2,2] × 3, [1,2,2]
Convolutions	2 encoder convolutions/stage; 2 decoder convolutions/stage
Normalisation/activation	InstanceNorm3d (eps 1 × 10^−5^, affine = True); LeakyReLU
Loss	Default nnU-Net Dice + cross-entropy compound loss with deep supervision
Optimiser/scheduler	SGD, initial LR 0.01, momentum 0.99, Nesterov = True, weight decay 3 × 10^−5^; polynomial LR schedule
Foreground oversampling	0.33; default non-probabilistic oversampling
Training schedule	1000 epochs; 250 training and 50 validation iterations/epoch
Manual tuning range	None; standard nnU-Net auto-configuration/default trainer
Software	Python 3.12.3; nnU-Net v2.6.4; PyTorch 2.8.0+cu126

**Table 3:** ATLAS out-of-fold performance stratified by released contrast-phase metadata.

Phase	*n*	Mean Dice (95% CI)	Median Dice (IQR)	Dice ≥ 0.50
Arterial	33	0.4869 (0.3774–0.5943)	0.6059 (0.1584–0.7093)	19/33 (57.6%)
Portal venous	10	0.6207 (0.3985–0.8094)	0.7484 (0.5450–0.8671)	8/10 (80.0%)
Delayed	8	0.5614 (0.3009–0.8071)	0.6702 (0.2800–0.9175)	5/8 (62.5%)
Phase unknown	7	0.6052 (0.4449–0.7601)	0.6352 (0.3837–0.7953)	4/7 (57.1%)
Non-contrast	2	0.4444 (0.0000–0.8887)	0.4444 (0.2222–0.6665)	1/2 (50.0%)

**Table 4:** External MRI positive-case performance by contrast phase.

Cohort	Phase	*n*	Mean Dice (95% CI)	Any-Overlap Detection	Complete Misses
LiverHccSeg	Arterial	14	0.6460 (0.4640–0.8077)	12/14 (85.7%)	2/14
LiverHccSeg	Portal-venous	14	0.6730 (0.4871–0.8262)	12/14 (85.7%)	2/14
LiverHccSeg	Delayed	14	0.6535 (0.4806–0.8087)	14/14 (100%)	0/14
LiverHccSeg	Native	14	0.3490 (0.1600–0.5507)	8/14 (57.1%)	6/14
OpenSwissHCC	Arterial	63	0.3388 (0.2497–0.4296)	35/63 (55.6%)	28/63
OpenSwissHCC	Venous	57	0.1267 (0.0603–0.2033)	16/57 (28.1%)	41/57
OpenSwissHCC	Delayed	58	0.1810 (0.1040–0.2648)	16/58 (27.6%)	42/58
OpenSwissHCC	Native	42	0.0814 (0.0254–0.1518)	10/42 (23.8%)	32/42

**Table 5:** OpenSwissHCC all-132 external safety summary.

Phase	Positive Mean Dice	Positive Detection	Specificity (69 HCC−)	FP Scan Rate	FP Components	Mean FP Volume/FP Scan (mL)
Arterial	0.3388 (0.2497–0.4296)	35/63 (55.6%)	46/69 (66.7%)	33.3%	57	227.36
Venous	0.1267 (0.0603–0.2033)	16/57 (28.1%)	56/69 (81.2%)	18.8%	24	184.80
Delayed	0.1810 (0.1040–0.2648)	16/58 (27.6%)	55/69 (79.7%)	20.3%	32	97.34
Native	0.0814 (0.0254–0.1518)	10/42 (23.8%)	35/69 (50.7%)	49.3%	59	135.64

**Table 6:** OpenSwissHCC lesion/component matching sensitivity (greedy assignment; Hungarian assignment was identical at all thresholds).

Phase	Criterion	TP	FP	FN	Precision	Recall	F1
Arterial	Any overlap	38	38	51	50.0%	42.7%	46.1%
Arterial	IoU ≥ 0.01	37	39	52	48.7%	41.6%	44.8%
Arterial	IoU ≥ 0.05	36	40	53	47.4%	40.4%	43.6%
Arterial	IoU ≥ 0.10	35	41	54	46.1%	39.3%	42.4%
Venous	Any overlap	18	10	67	64.3%	21.2%	31.9%
Venous	IoU ≥ 0.01	15	13	70	53.6%	17.6%	26.5%
Venous	IoU ≥ 0.05	15	13	70	53.6%	17.6%	26.5%
Venous	IoU ≥ 0.10	12	16	73	42.9%	14.1%	21.2%
Delayed	Any overlap	18	8	69	69.2%	20.7%	31.9%
Delayed	IoU ≥ 0.01	18	8	69	69.2%	20.7%	31.9%
Delayed	IoU ≥ 0.05	18	8	69	69.2%	20.7%	31.9%
Delayed	IoU ≥ 0.10	17	9	70	65.4%	19.5%	30.1%
Native	Any overlap	9	27	46	25.0%	16.4%	19.8%
Native	IoU ≥ 0.01	8	28	47	22.2%	14.5%	17.6%
Native	IoU ≥ 0.05	5	31	50	13.9%	9.1%	11.0%
Native	IoU ≥ 0.10	5	31	50	13.9%	9.1%	11.0%

**Table 7:** OpenSwissHCC subject-level HCC size-burden stratification.

Phase	All Documented HCC < 20 mm	At Least One Documented HCC ≥ 20 mm
Arterial	7/18 detected (38.9%); mean Dice 0.202	27/41 (65.9%); mean Dice 0.428
Venous	2/17 (11.8%); mean Dice 0.005	14/40 (35.0%); mean Dice 0.178
Delayed	2/16 (12.5%); mean Dice 0.050	14/40 (35.0%); mean Dice 0.242
Native	1/12 (8.3%); mean Dice 0.0004	8/27 (29.6%); mean Dice 0.111

**Table 8:** All six paired OpenSwissHCC phase comparisons in complete four-phase cases (*n* = 38).

Contrast	Mean Dice Difference	95% Bootstrap CI	Holm-Adjusted *p*
Arterial–Venous	+0.186	0.071 to 0.307	0.0382
Arterial–Delayed	+0.087	−0.053 to 0.231	0.6129
Arterial–Native	+0.239	0.120 to 0.362	0.0141
Venous–Delayed	−0.099	−0.175 to −0.034	0.0382
Venous–Native	+0.053	−0.033 to 0.146	0.6129
Delayed–Native	+0.151	0.058 to 0.255	0.0388

**Table 9:** Primary paired Dataset804 (venous-only) versus Dataset805 (arterial-plus-venous) OpenSwissHCC ablation.

Outcome	Venous Only	Arterial + Venous	Difference	Paired Test *p*
HCC+ mean Dice	0.2269	0.3705	+0.1435 (95% CI +0.0520 to +0.2343)	0.00223
HCC+ median Dice	0.0249	0.4245	+0.3996 (bootstrap)	-
HCC+ any-overlap detection	52.4%	68.3%	+15.9 percentage points	0.0639 (exact McNemar)
Aggregate voxel sensitivity	0.1580	0.3518	+0.1939	0.00485
HCC−correct-empty specificity	30.4%	42.0%	+11.6 percentage points	0.2005 (exact McNemar)
Mean FP voxels/negative scan	640.3	209.8	−430.5 (95% CI −749.2 to −168.1)	0.0394

**Table 10:** Exploratory MRI-to-CT stress-test performance.

Cases	Mean Dice (95% CI)	Median Dice	GT Components	Precision	Recall	F1	FP/Case
97	0.0286 (0.0100–0.0522)	0.0000	847	5.3%	2.2%	3.2%	3.48

**Table 11:** Structured comparison with selected MRI liver/HCC segmentation studies.

Study	Data/Design	Model	Reported Tumour Result	Interpretive Context
Bousabarah et al. [5]	174 patients; 3-phase DCE-MRI	2D U-Net	Mean Dice 0.68	Multiphasic within-study evaluation
Hansch et al. [6]	Late-phase DCE-MRI; 19 annotated test cases	Anisotropic 3D U-Net	Mean Dice 0.74; lesion F1 0.59	Small-lesion detection remained difficult
Zheng et al. [7]	Dynamic DCE-MRI with 4D information	3D CNN + ConvLSTM	Dice 0.825 +/− 0.077	Temporal multiphasic model
Luo et al. [8]	382 patients/466 lesions; independent/external testing	Modified multiphasic U-Net ensemble	Dice 0.81 +/− 0.11	Larger pathologically confirmed cohort
Raab et al. [9]	458 multiphase scans	nnU-Net variants and Swin UNETR	Lesions remained most difficult target	Phase-rich cross-scanner development
Karabağ et al. [10]	Public ATLAS; five-fold validation plus held-out testing	3D nnU-Net and comparators	Five-fold 0.915 +/− 0.016; held-out 0.892	Different task/protocol/aggregation; not directly equivalent
Present study	ATLAS OOF + phase-wise external stress tests + OpenSwissHCC ablation	Standard 3D nnU-Net v2	Internal mean 0.5315; external domain-sensitive	Audited targets, all-negative safety, matching sensitivity and paired ablation

**Table 12:** Protocol reconciliation between the present ATLAS benchmark and Karabağ et al. [10].

Domain	Present Study	Karabağ et al. [10]	Implication
ATLAS cases	60 public ATLAS training cases; every case appears once in OOF validation	Public ATLAS; paper describes five-fold evaluation and also a held-out test design	Case-partition relationship is not identical/fully recoverable from publication
Target	Binary tumour only; liver/background mapped to 0	Liver and tumour segmentation outputs reported	Task construction differs
Folds	5 patient-level folds, 48 train/12 validation each; assignments archived	Exact five-fold patient assignments not published	Fold composition cannot be matched unambiguously
Preprocessing	nnU-Net v2.6.4; spacing 3.0 × 1.0417 × 1.0417 mm; Z-score; archived plans	Exact nnU-Net version/plans and preprocessing settings insufficiently specified	Configuration equivalence cannot be assumed
Checkpoint/ensemble	Terminal/final checkpoint per fold for OOF; five-fold ensemble externally	Exact checkpoint selection/ensemble policy insufficiently specified	Potential source of performance difference
Post-processing	Raw and standard nnU-Net post-processed OOF summaries numerically identical; no manual threshold, component filtering, size filter or smoothing	Tumour-specific post-processing not sufficiently specified for direct reproduction	Post-processing does not explain our internal result; identical comparator processing cannot be enforced
Aggregation	Mean per-case OOF Dice 0.5315; pooled-voxel Dice 0.6231	Five-fold tumour Dice 0.915 +/− 0.016; separate held-out tumour Dice 0.892	Absolute gap vs. 0.915: 0.3835 per-case/0.2919 pooled; vs. 0.892: 0.3605/0.2689
Reproduction conclusion	Protocol audited and permanently released with versioned repository	Published details insufficient for unambiguous identical reconstruction	Aggregation explains only part of the gap; residual discrepancy remains incompletely explained

## Data Availability

ATLAS, LiverHccSeg, OpenSwissHCC and HCC-TACE-Seg remain available from the original repositories cited in References [14,15,16,17], under their respective access and licensing terms. Source medical images are not redistributed. Reproducibility materials supporting the principal analyses—including code, patient-level fold assignments, derived manifests, HCC-reference reconstruction scripts, nnU-Net configurations and trained checkpoints, prediction manifests, case-level metrics, statistical-analysis outputs, provenance documentation and reviewer-requested reanalyses—are permanently archived at Zenodo (version 1.0.0), DOI: 10.5281/zenodo.22228909. The corresponding source code is available at https://github.com/gggsgourakis-sketch/HCC-MRI-Segmentation-Reproducibility (accessed on 1 September 2026).

## Abbreviations

ASSD, average symmetric surface distance; CI, confidence interval; CT, computed tomography; DCE-MRI, dynamic contrast-enhanced MRI; FN, false negative; FP, false positive; GT, ground truth; HCC, hepatocellular carcinoma; HD95, 95th-percentile Hausdorff distance; IoU, intersection over union; MRI, magnetic resonance imaging; OOF, out-of-fold; T1w, T1-weighted; and TP, true positive; ATLAS, A Tumor and Liver Automatic Segmentation; ReLU, rectified linear unit; SGD, stochastic gradient descent; CNN, convolutional neural network; UNETR, U-Net Transformer; ConvLSTM, convolutional long short-term memory.
